# EU Nitrates Directive, from theory to practice: Environmental effectiveness and influence of regional governance on its performance

**DOI:** 10.1007/s13280-019-01197-8

**Published:** 2019-05-21

**Authors:** Arianna Musacchio, Viviana Re, Josep Mas-Pla, Elisa Sacchi

**Affiliations:** 1 Department of Earth and Environmental Sciences, University of Pavia, Via Ferrata 9, 27100 Pavia, Italy; 2 Institut Català de Recerca de l’Aigua, 17003 Girona, Spain; 3 Institut Català de Recerca de l’Aigua, 17003 Girona, Spain

**Keywords:** Agriculture, Contamination trends, Knowledge transfer, Lombardy plain, Social network analysis, Socio-hydrogeology

## Abstract

Despite the European Nitrate Directive (ND) being issued almost 30 years ago, groundwater nitrate contamination is still a serious threat to ecosystems and human health. In one of the areas most affected by nitrates, the Lombardy Plain (Italy), the effectiveness of the ND and the capacity of governance to support its application correctly was assessed using a socio-hydrogeological approach. Nitrate trends over 11 years show that most regions present steady or increasing concentrations, highlighting how contamination can affect previously impaired situations and supposedly resistant and resilient aquifers. Stakeholder network analysis reveals that the governance framework does not support knowledge dissemination and changes in farmers’ attitudes, hindering water quality improvements. Nitrogen input needs to be reduced and manure relocation monitored. The local governance scale has a key role in enhancing ND dissemination. Reports to the EU Commission should integrate multi- or interdisciplinary evaluation of trends, including governance dynamics, alongside hydrochemical information.

**Electronic supplementary material:**

The online version of this article (10.1007/s13280-019-01197-8) contains supplementary material, which is available to authorized users.

## Introduction

For almost 30 years, the Nitrate Directive (ND; 91/676/EEC) has been the main European reference for the protection of water threatened by over-exploitation of agricultural land and the resulting nitrate contamination. The ND was issued in 1991 to “protect water quality across Europe by preventing nitrates from agricultural sources polluting ground and surface waters and by promoting the use of good farming practices” (EU Commission 1991). Accordingly, Member States were asked to designate Nitrate Vulnerable Zones (NVZs), namely areas likely to contribute to surface or ground water contamination of a minimum of 50 mg L^−1^ of nitrate (NO_3_^−^). Within the NVZs, specific mandatory protection measures had to be adopted by farmers and a limit of 170 kg ha^−1^ year^−1^ of nitrogen (N) from organic manure was established. Furthermore, within the non-vulnerable zones (nNVZs), Member States had to propose a set of measures to be implemented on a voluntary basis, mainly regarding the periods and weather conditions for fertiliser application. The ND is also one of the Statutory Management Requirements that European farmers are obliged to respect in order to receive the subsidies provided for the cross-compliance system of the Common Agriculture Policy. Individual benefits are reduced proportionally to any detected noncompliance.

After almost three decades, there is no significant reduction in groundwater nitrate contamination, and agriculture is still the main source of nitrate pollution in Europe (EU 2010). About half of the European monitoring stations show no significant change in nitrates, and 26.6% of them present increasing trends (EU Commission 2013). Average concentrations in aquifers are the same as in 1992, revealing that efforts are still required to restore groundwater quality (EEA 2015).

Clearly, the time gap between the implementation of conservation measures and the first measurable improvements is a common feature of nonpoint source pollution in aquifers. However, unmet expectations may generate growing discontent and frustration (Meals et al. 2010). Understanding the causes of this delay and determining its duration is therefore a crucial task for effective and durable water resource management.

The achievement of appreciable declines in groundwater nitrate concentrations is influenced by both intrinsic factors (i.e. the biogeochemical, pedological and hydrogeological characteristics of the system) and multiscale groundwater governance. Recently it was observed that failure in groundwater management is often the result of an inadequate governance configuration, rather than the lack of knowledge related to aquifer vulnerability or hydrogeological dynamics (Foster and Garduno 2013; Garrick et al. 2017).

By investigating the case of an emblematic area for the nitrate issue in Europe, we assess (i) whether groundwater contamination has decreased since the ND has been applied, and (ii) whether regional groundwater governance supports the application of the ND correctly, with the aim of looking for possible links between nitrate trends and governance actions. To address our aims, we begin by conducting a contamination trend analysis in the Lombardy plain (northern Italy). This is one of the main European basins in terms of groundwater storage (BGR/UNESCO 2008) but it is also subject to one of the highest nitrate inputs in Europe (Eurostat 2012). Then, we identify stakeholders involved in groundwater governance and ND implementation, and the socio-relational dynamics between them through a social network analysis. Finally, we discuss management and governance opportunities.

## Theoretical framework

The concept of governance refers to “the range of political, social, economic and administrative systems that are in place to regulate development and management of water resources and provisions of water services at different levels of society” (Rogers and Hall 2003). Unlike management, in its broadest sense governance includes the complexity of the regulatory processes that result from the interaction between the different actors who help to define the legal framework and then implement the environmental policy and its tools (Pahl-Wostl 2009). Therefore, governance is an ongoing process in which multiple actors on different scales, with multiple purposes and priorities, interact more or less directly through formal and informal relationships.

To understand the governance structure and the main relational patterns influencing ND application, we adopted a social relational approach using social network analysis (Bodin and Prell 2011). According to this approach, investigating how the actors contribute and influence natural resource governance requires a more systemic perspective, namely exploring how the actors are framed in the wider social context, instead of considering them separately. The sociological theories underpinning the social relational approach (Sawyer 2002) assume that the cultural, economic and political properties of a system are not the mere sum of the attributes of its components (i.e. the actors and their actions); rather, they are new emerging properties, determined by both the relational structure and the way the actors are tied to and positioned in the social system. In other words, patterns of relationships and configurations of governance can constrain or promote different attitudes and behaviours of both the actors involved and the system as a whole. From a methodological perspective, the social relational approach is implemented by means of the social network analysis, allowing us to understand the social system in its complexity by visualising it as a graph. The nodes in the graph represent the actors, and the links the relationships between them. Qualitative and quantitative analysis of this graph provides insights into the influence of governance structure on observed behaviours and patterns.

As regards groundwater, the implementation of multidisciplinary investigations in hydrogeological science is still far from being common practice (Re and Misstear 2017) and the increase in the use of social science keywords is not reflected in the way hydrogeological research is carried out (Barthel and Seidl 2017). In this framework, the socio-hydrogeological approach was recently formalised with the general aim of fostering “the inclusion of social dimension in the hydrogeological investigations” (Re 2015). According to this approach, hydrogeologists should consider the mutual relationships between people and groundwater, starting by identifying both the actors affected (directly or indirectly) by the groundwater system and the socio-economic factors hindering the effective implementation of good management practices. Although social network analysis was shown to be an effective method for these aims, its application is quite a novelty in hydrogeological assessments. To our knowledge it was only used in two studies that explicitly focused on groundwater governance, both of which were carried out in rural developing regions (Kuzdas et al. 2015; Tringali et al. 2017).

## Study area

**Fig. 1:**
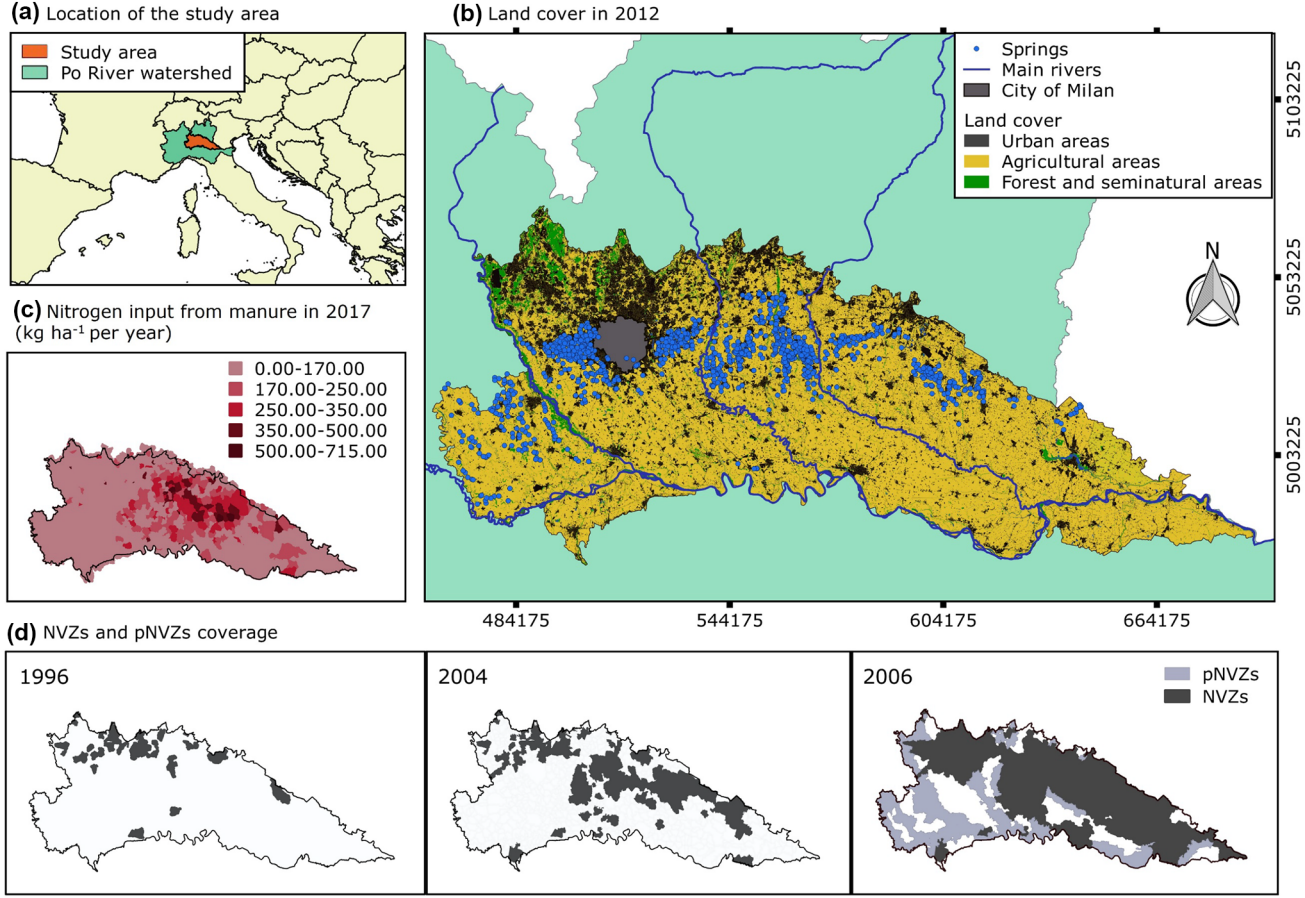
**a** Location of the study area, **b** land cover in 2012, **c** Nitrogen input from manure in 2017 in kg ha^−1^ per year, **d** increase in coverage of Nitrates Vulnerable Zones (NVZs) and partial Nitrates Vulnerable Zones (pNVZs). Coordinates refer to WGS 1984 e UTM Zone 32 N projection

The Lombardy plain belongs to the Po River watershed (Fig. 1a), the largest in Italy (71 057 km^2^), hosting one of the largest multilayer aquifers in Europe. Intensive agriculture, industries and human settlements make the Po River watershed a strategic area for the Italian economy, while generated and transported N loads have a recognised impact on Mediterranean ecosystems (Bartoli et al. 2012, and references therein).

The Lombardy plain (13 700 km^2^) accounts for about 18% of the Po catchment and 30% of the Po Plain area. The alluvial sequence, made of gravel and sand with interbedded clay layers, creates a multilayer aquifer system of relevant extension (more than 11 200 km^2^ and up to 500 m deep) with three main hydrogeological units: the shallow, intermediate and deep aquifers (Éupolis Lombardia 2015).

In the Lombardy plain, urban and industrial areas cover about 22% of the land and include the city of Milan and several important industrial districts. The remaining area is devoted to corn and wheat (70%), and rice (11%) cultivation. NVZs presently cover about 56% of the plain (Fig. 1b, c). These have been designated since 1996 (5.7%) and extended by subsequent additions, during a long negotiation between the EU and Regional Authorities (Martinelli et al. 2018). Vulnerability was defined on a municipal scale, and municipalities only partially subject to NVZ regulations were classified as partially Nitrate Vulnerable Zones (pNVZ; Fig. 1d).

All previous studies addressing nitrate contamination (e.g. Sacchi et al. 2013; Stevenazzi et al. 2015; Musacchio et al. 2019), conclude that it is due to a combination of (i) intrinsic characteristics (i.e. high infiltrability), (ii) high anthropogenic pressure of both agricultural and civil origin and (iii) agricultural practices (i.e. high recharge by irrigation returns). Due to the acknowledged presence of both diffuse and point pollution sources, drinking wells in the shallow aquifers have been progressively disconnected in recent decades and water abstraction redirected to the intermediate and deeper aquifers.

## Materials and Methods

### Nitrate contamination values and trends

Current nitrate levels were assessed using monitoring data collected by the Regional Agency for Environmental Protection (ARPA) from 258 wells (168 tapping the shallow aquifers, 58 the intermediate and 31 the deeper aquifers; ARPA Lombardy 2018). The mean nitrate concentrations detected in each well in 2016 were calculated and a Wilcoxon rank-sum test was performed to determine significant differences between aquifers (Helsel and Hirsch 2002).

The nitrate trend analysis used ARPA monitoring network data from 2006 (i.e. the year the ND was fully implemented on a regional scale) to 2016 (ARPA Lombardy 2018). The non-parametric Mann–Kendall test was applied to statistically detect significant monotone trends, using a 95% significance level, and to categorise the wells as decreasing, increasing or non-detected trends. The latter corresponds to a non-statistically significant trend. Available data were selected, based on Hirsch et al. (1991), and a list of suitable wells obtained (additional details in the Electronic Supplementary Materials, ESM). For detected trends, the Sen slope estimator was calculated to estimate the magnitude, namely nitrate increase or decrease (in mg L^−1^ per year) (Sen 1968). The Wilcoxon rank-sum test was applied to explore significant differences in trend magnitudes (i) between aquifers and (ii) between four concentration classes (< 10; 10–25; 25–50; > 50 mg L^−1^) (Eurostat 2012). Finally, the distribution of trends in NVZs, pNVZs and nNVZs was explored.

### Governance framework

To identify governance structure and dynamics, a social network analysis was carried out using the participative network mapping tool called “Net-map” (Schiffer and Hauck 2010), which obtains both network data and qualitative descriptions of relationships and roles in the network. Qualitative data, also called “network narratives”, provide information on intersubjective meanings attributed to network components and shared or predominant perceptions (Hauck et al. 2015; Fuhse and Mutzel 2011). Between October 2016 and June 2017, we carried out in-depth focus groups with five groups of key informants: (i) authorities (members of the Regional Directorates for Agriculture and for Environment, and of ARPA), (ii) farmers, (iii) breeders, (iv) organisations (representatives of a farmers’ trade union and a Water Consortium), (v) scientists actively involved in research projects. Each group was interviewed separately in order to avoid possible bias due to power differences or intimidation. During the meetings, the interviewees were asked to draw the social network involved (directly or indirectly) in nitrate contamination. Each network summarises the information related to: (i) the actors involved in the studied issue, (ii) their relationships (in terms of control and authorisation, exchange of advice, technical information, money, conflicts; Table S1), (iii) their influence, according to the interviewees’ perception (evaluated on an influence-value scale, from 1-lower to 5-higher). The five networks were then merged and the average influence levels of each actor were calculated (more details in ESM). By virtue of both the quantitative and qualitative nature of collected data, structural and content analyses were combined (Hauck et al. 2016). The structural role of each actor was evaluated by measuring degree centrality (i.e. the total number of ingoing and outgoing links of an actor; Wasserman and Faust 1994) and betweenness centrality (i.e. how many times an actor is found on the shortest path connecting other actors who are otherwise disconnected; Freeman 1978). Actors with a degree centrality, betweenness centrality or influence higher than the 75th percentile were considered as the most central, connecting or influent actors. Average influence, centrality measures and description of actors in network narratives were compared to detect discrepancies between structural and perceived roles. As regards content analysis, a description of each kind of link was obtained by using recordings and transcriptions from focus groups to define the way that relationships support or hamper ND application according to stakeholders’ opinions. The focus groups revealed a distinct difference in the perception of meanings and network components between groups of key informants; therefore, the analysis was enhanced by measuring (i) how many times each actor was mentioned by the different groups of key informants, (ii) the percentage of actors listed by each focus group and included in the merged network and (iii) the difference between the minimum and maximum value of influence assigned to each actor by different focus groups. Actors mentioned less than 3 times were considered scarcely perceived. Differences in influence of three or more were considered as significant in terms of divergence in perception between groups of key informants. Consistently, the descriptions of relationships were integrated with data on differences in perception of structure and meanings associated to each kind of link.

## Results

### Current values of nitrates and concentration trends

**Fig. 2:**
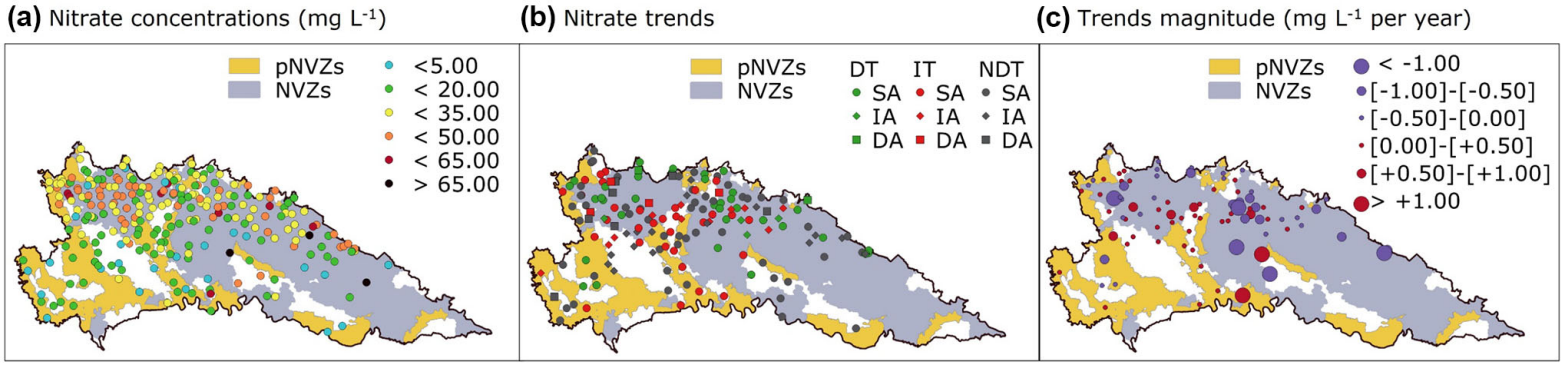
**a** Mean nitrate concentrations in 2016 (ARPA monitoring data); **b** trends of nitrate concentrations in the three groups of aquifers, from 2006 to 2016 (*SA* shallow aquifers, *IA* intermediate aquifers, *DA* deeper aquifers); **c** magnitude of nitrate trends. Nitrates Vulnerable Zones (NVZs) and partially Nitrates Vulnerable Zones (pNVZs) are reported

Results highlight significant differences between the nitrate levels in the shallow aquifers compared to the intermediate and deeper ones (*W*_shallow-intermediate_ = 3426, *p *= 0.002; *W*_shallow-deeper_ = 3426, *p *= 0.007; mean_shallow_ = 26.5 mg L^−1^, mean_intermediate_ = 18.5 mg L^−1^, mean_deeper_ = 17.7 mg L^−1^; Fig. 2a). 43.4% of the wells are currently above the “concern” threshold of 25 mg L^−1^ (Eurostat 2012), and 4.7% are above the statutory limit of 50 mg L^−1^.

**Fig. 3:**
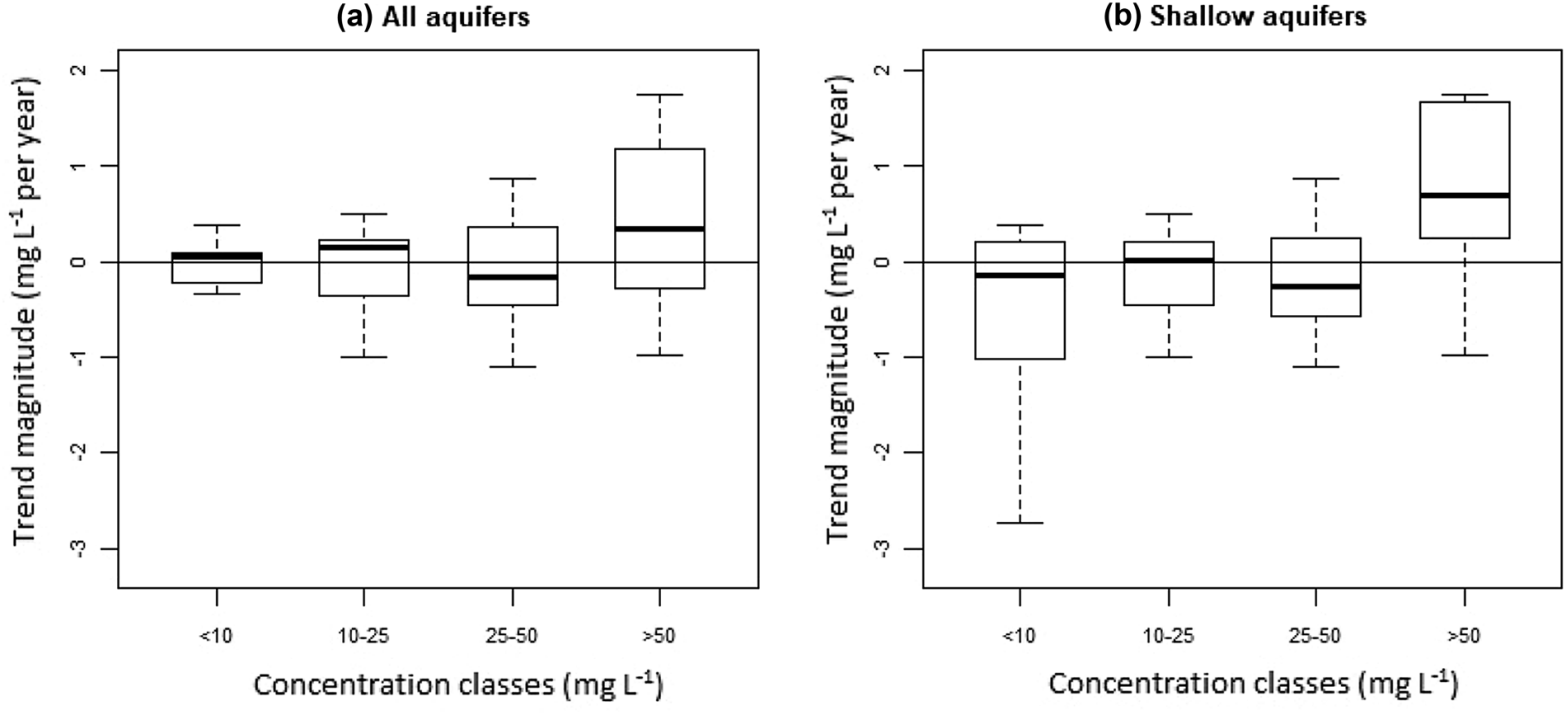
**a** Trend magnitudes for nitrate concentration classes; **b** trend magnitudes for nitrate concentration classes in shallow aquifers

Out of the 162 wells suitable for trend analysis (118 in shallow, 31 in intermediate and 13 in deeper aquifers), 46.3% are classified as non-detected, 28.4% as increasing and 25.3% as decreasing trends (Fig. 2b; Table 1). Overall, in the three aquifers, approximately half of the wells are classified as non-detected trends (Table 1). Regarding the trend magnitudes, the annual increase and decrease ranges between + 0.05 and + 1.7, and between − 0.08 and − 3.4 mg L^−1^, respectively. On average, these variations are higher in wells tapping shallow aquifers (Table 1), although these differences are not statistically significant. Trend magnitudes show both higher average and higher maximum annual concentration changes in the class with concentrations > 50 mg L^−1^ (mean: 0.41; max: 1.74; mean_shallow_: 0.68; max_shallow_: 1.74 mg L^−1^ per year Fig. 3), although differences between classes are not significantly different. Decreasing average values are found in the class 25–50 mg L^−1^ in both analysed groups (mean = − 0.15; mean_shallow_ = − 0.24 mg L^−1^). A higher proportion of increasing trends was detected in the municipalities currently classified as nNVZs and pNVZs, compared to those currently classified as NVZs. These wells generally have low concentrations and are mostly located in the southern sector (Fig. 2). The trend magnitude of increasing wells located in nNVZ is on average higher than that of wells located in NVZ (Table 2; Fig. 2b, c).

**Table 1:** The number (%) of wells having non-detected, increasing and decreasing trends, in each group of aquifers, and the minimum, mean and maximum trend magnitudes

Aquifers	No.	Wells			mg L^−1^ per year in increasing trends			mg L^−1^ per year in decreasing trends		
		% Non-detected	% Increasing	% Decreasing	Min	Mean	Max	Min	Mean	Max
Shallow	118	44.1	26.3	29.7	0.1	0.4	1.7	− 0.1	− 0.7	− 3.4
Intermediate	31	51.6	35.5	12.9	0.1	0.3	0.8	− 0.1	− 0.3	− 0.8
Deeper	13	53.8	30.8	15.4	0.1	0.2	0.4	− 0.8	− 0.2	− 0.04

**Table 2:** The number of wells (%) having non-detected, increasing or decreasing trends within nitrates vulnerable zones (NVZ), partially vulnerable zones (pNVZ) and non-vulnerable zones (nNVZ), and their average magnitude of trends

Vulnerability	No.	Wells			Trend magnitude (mg L^−1^ per year)	
		% Non-detected	% Decreasing	% Increasing	Decreasing	Increasing
NVZ	92	40.2	35.9	23.9	− 0.60	0.38
pNVZ	29	55.2	13.8	31.0	− 0.32	0.25
nNVZ	41	53.7	9.8	36.6	− 1.05	0.43

### Governance framework

**Fig. 4:**
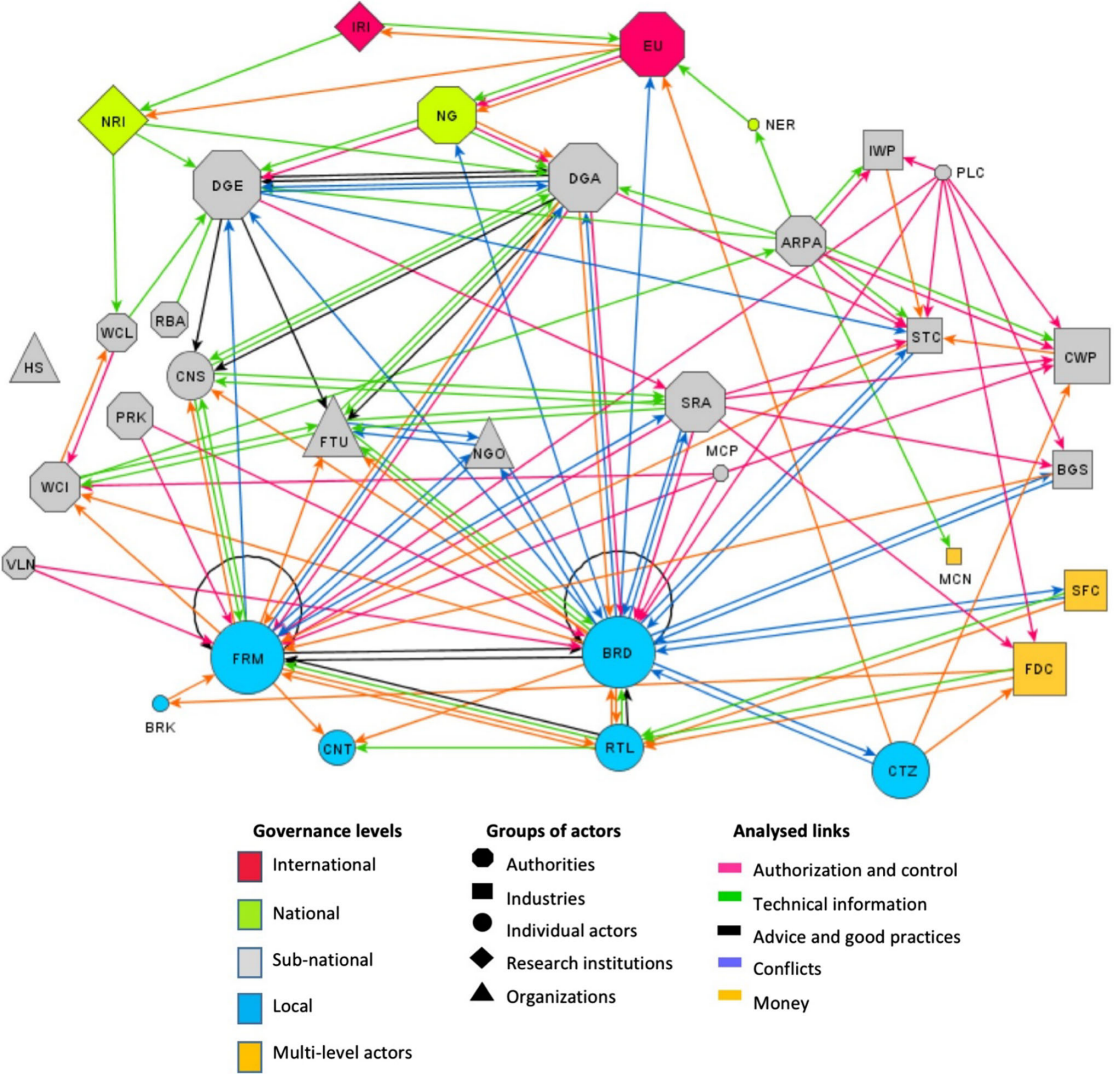
Final INM representing the governance framework. The size of the nodes corresponds to the perceived influence of each actor. List of acronyms used in the map in alphabetical order: *ARPA* Regional Agency for Environmental Protection, *BGS* biogas and compost plants, *BRD* breeders, *BRK* brokers, *CNS* agricultural consultants, *CNT* agricultural contractors, *CTZ* non-farm residents, *CWP* civil wastewater treatment plants, *DGA* Regional Directorate for Agriculture, *DGE* Regional Directorate for Environment, *EU* European Commission, *FDC* food companies, *FRM* farmers, *FTU* farmers’ trade unions, *HS* high schools, *IRI* international research institutes, *IWP* industrial wastewater treatment plants, *MCN* agricultural machinery manufacturers, *MNC* municipalities, *NER* National Institute for Environmental Protection and Research, *NG* national government, *NGO* environmental NGO, *NRI* national research institutions, *PLC* environmental police, *PRK* parks, *RBA* Po River Basin Authority, *RTL* agricultural retailers, SFC seed, fertilisers, animal feed companies, *SRA* sub-regional administrations, STC sludge treatment companies, *VLN* environmental volunteers, *WCI* water consortia (irrigation), *WCL* water consortia (lakes)

The governance network supporting the application of ND includes 33 actors, distributed across the four main governance levels (Fig. 4; Table 3). Among them, 10 were not mentioned more than twice (Table 4). The median percentage out of 33 actors listed by each focus group was 54.5% (Table S2). 27 actors show relevant differences in influence values (Table 4). Only farmers, breeders and national research institutions were highly mentioned and, at the same time, similarly perceived by interviewees in terms of influence. 11 out of the 33 actors were shown to be more relevant than the others (Table 4); except for the agricultural retailers, all of them were mentioned at least four times. Seven of these relevant actors were both highly influential and central or connecting, and their roles and responsibilities were also described by different groups of key informants in the same way:

**Table 3:** Network composition in terms of groups of actors and kind of links

Groups of actors	%	Links	%
Authorities	42.4	Technical information	27.5
Individual actors	21.2	Authorisation and control	23.2
Industries	21.2	Money flows	21.7
Organisations	9.1	Conflicts	18.8
Research institutions	6.1	Advice and best practices	8.7

**Table 4:** Results concerning the average influence of the actors, the betweenness and degree centrality, the number of times the actors were mentioned and the difference between the maximum and minimum value of influence assigned to them by different groups of key informants. Asterisks identify the most relevant actors, i.e. having highest values of influence, centrality or betweenness, based on 75th percentile. For these actors, asterisks are reported only on those measures which value result higher than the 75th percentile

Actor	Acronym	Influence	Degree centrality (%)	Betweenness centrality (%)	Times mentioned	Max–min influence
Farmers*	FRM	4.2*	10.5*	10.5*	5	2
Breeders*	BRD	4.2*	13.4*	25.0*	5	2
Regional DG Agriculture*	DGA	3.8*	8.0*	6.9*	5	4
Regional DG Environment*	DGE	3.6*	5.8*	6.0*	5	4
Sub-regional administrations*	SRA	2.8*	5.1*	6.4*	5	3
European Commission*	EU	3.2*	3.3	6.3*	4	4
Farmers’ trade unions*	FTU	3*	5.1*	3.6	5	4
National research institutions*	NRI	4.0*	1.8	3	5	2
Agricultural retailers*	RTL	1.8	4.7*	1.9	3	5
Regional environmental protection agency*	ARPA	2	4	7.1*	4	4
Water consortia (irrigation)*	WCI	2	2.9	10.6*	5	3
Agricultural consultants	CNS	1.8	3.6	0.1	3	5
Agricultural contractors	CNT	1	1.1	0	2	4
Agricultural machinery manufacturers	MCN	0.2	0.4	0	1	1
Biogas and compost plants	BGS	1.2	1.8	0	3	4
Brokers	BRK	0.2	0.7	0.1	1	1
Non-farm residents	CTZ	2.6	1.8	0.9	4	5
Civil wastewater treatment plants	CWP	2.4	2.5	0.1	3	5
Environmental NGO	NGO	2	2.2	0	3	4
Environmental Police	PLC	0.2	2.5	0	1	1
Environmental volunteers	VLN	0.8	0.7	0	1	4
Food companies	FDC	2.2	2.2	2.3	4	4
High schools	HS	2	0	0	2	5
Industrial wastewater treatment plants	IWP	1.2	1.4	0	2	5
International research institutes	IRI	2	1.1	0	3	5
Municipalities	MNC	0.2	1.4	0	3	4
National government	NG	2.6	3.3	2.4	3	4.5
National Institute for Environmental Protection and Research	NER	0.1	0.7	0.4	2	4
Parks	PRK	1.7	0.7	0	3	4
Po River Basin Authority	RBA	1	0.4	0	2	4
Seed, fertilisers, animal feed companies	SFC	1.4	1.4	0	3	3
Sludge Treatment Companies	STC	1	4	4.6	1	5
Water consortia (lakes)	WCL	1.2	1.4	1.5	3	4

- Farmers and breeders are the most influential actors according to key informants’ perception (i.e. given the potential impact of agricultural and breeding activities on water quality) and centrality measures.
- Agricultural trade unions (hereafter referred to as “trade unions”) are the link between authorities and farmers in terms of information and administrative flows.
- European, regional and sub-regional authorities have a key role in defining and applying the ND, supplying Common Agriculture Policy subsides, coordinating and implementing controls. The European Commission is not central; its high perceived influence is actually confirmed by its betweenness value, consistent with its connecting role described by key informants. The lack of a high centrality degree for this actor could be expected considering the regional scale of ND application we focused on.

The high influence that key informants uniformly assign to national research institutions is not confirmed by centrality measures (Table 4). The central role of retailers and the connecting capacity of ARPA and Irrigation Water Consortia do not correspond to high perceived influence. Centrality measures reveal that trade unions do not have a highly connecting capacity, though their influence is mainly associated to this function in interviewees’ opinion.

As regards relationships, 138 links were identified (Table 3). In the light of the similar role played by farmers and breeders, from now on we will refer to both as “farmers”, unless otherwise indicated. We will also refer to information and advice flows jointly, as “knowledge flow”. The network narratives highlight the following:

- During all focus groups, difficulties in fully mastering both authorisation and control ties (more details in ESM; Fig. S1) and the tasks of several authorities (i.e. municipalities, provinces, ARPA, River Basin Authority and Water Consortia) were detected. All interviewees reported that control-based strategies represent the only real tool to guarantee the adoption of sustainable practices by farmers but, at the same time, it is extremely difficult to perform systematic and widespread controls due to the number of farms and the associated costs. Farmers observed that recurrent controls may be a life-long learning opportunity if associated to structured capacity building and to a mutual trust relationship between farmers and authorities.
- European direct payments associated to the ND, research funds, costs for wastewater treatment and economic relations between the agricultural and industrial sectors are the main money links (Fig. S2). Only authorities and farmers showed a detailed knowledge of the money flows that farmers are involved in, despite their relevance in defining farmers’ decision-making. To comply with the limits defined by the ND, breeders usually give superfluous manure to farmers. This manure relocation neither corresponds to money flows nor is tracked by authorities.
- Two knowledge flows are present: one based on a traditional knowledge-transfer approach (i.e. information is generated in science and transferred to farmers through authorities and trade unions; Fig. S3; Blackstock et al. 2010), the other connects the seed, food and fertiliser companies to farmers through retailers (Fig. S4). Authorities inform farmers about rules for ND application and periods in which fertilisation is forbidden or permitted, via official websites, newsletters and smartphone applications. Trade unions provide farmers with administrative support regarding ND and subsides. Occasionally they produce dissemination materials on ND. Authorities, researchers and organisations disregard the advice generated by the industrial sector. In general, a conceptual diversity emerges between groups of key informants with regards to the advice flow. Authorities, researchers and organisations see no or little difference between “information” and “advice”, as both aim to improve water quality. Farmers distinguished the two flows: they associate technical information with the compliance with the rules required to obtain subsides and the bureaucratic procedures (i.e. documents quantifying nitrate inputs, provided by farmers to authorities through trade unions); on the other hand, reliable advice that can change farm strategies is provided by neighbouring farmers and retailers. The retailers establish business relationships locally, based on direct meetings with farmers. In farmers’ opinion, frequency, informality and consistency with their production goals makes the relationships that occur on a local scale (i.e. with retailers and other farmers) stronger and relevant compared to those with the actors of other governance scales. Additionally, agricultural magazines as a trustworthy information source and agricultural high schools may have a key role in enhancing environmental awareness in the agricultural sector, according to farmers.
- Conflicts (Fig. S5) between farmers and authorities on a regional, national and European scale are mainly due to slurry or manure spreading and to ND restrictions; conflicts between farmers, non-farm residents and sub-regional authorities are mainly caused by smells produced by effluent spreading.

## Discussion

There has been overall stability in groundwater contamination in the Lombardy plain since the full implementation of the ND. Most wells in the three aquifers present no significant trends in nitrate concentrations. Current nitrate concentrations are not particularly alarming, often being lower than the threshold levels. Nevertheless, contamination is ongoing, as testified by increasing trends detected in about a third of the monitoring wells (Table 1). Moreover, current application of the ND is not sufficient either to reduce nitrate contamination in previously impaired areas, or to preserve the higher groundwater quality of resistant and resilient hydrogeological units. Indeed, the in-depth trend analysis revealed three critical issues:

(i) Increasing trends, mostly in wells that already exceed threshold values (50 mg L^−1^; Fig. 3).
(ii) Deteriorating groundwater quality in the supposedly protected intermediate and deeper aquifers, highlighted by both the high proportion of wells with increasing values and the magnitude of their trends (compared with those of the shallow aquifers). Intermediate and deeper aquifers are generally considered less vulnerable to contamination (Éupolis Lombardia 2015), and are extremely relevant as a strategic resource in the light of possible droughts driven by climate change and/or over-exploitation of shallow aquifers.
(iii) Increasing trends within the nNVZs located in the southern plain, which is generally more resilient. In this area, the intrinsic characteristics of the aquifers and the agricultural practices promote denitrification, i.e. the system’s ability to self-recover (Sacchi et al. 2013; Martinelli et al. 2018), which justifies the presence of extended nNVZs. Most wells with increasing trends are not located in areas characterised by recent urban sprawling or population growth (Stevenazzi et al. 2015), and their low concentrations (Fig. 2) do not suggest local phenomena of point-source pollution. Therefore, we can hypothesise that nitrogen input from agricultural activities is slowly contributing to exceeding the self-recovery capacity of the area. This excess may (paradoxically) be promoted by manure relocation adopted by breeders to comply with the ND.

As regards groundwater governance, the implementation of management practices is mainly based on the activity of a small number of relevant actors (i.e. farmers, trade unions, European, regional and sub-regional authorities), whose influence and roles are uniformly described and confirmed by the centrality measures (Table 4). Besides these few actors, the analysis reveals the divergence between the governance perceived and its structure, and the lack of a common vision on governance components and dynamics. The role of some actors (i.e. ARPA, retailers, Irrigation Water Consortia) with high centrality or betweenness values is not perceived, as indicated by their low perceived influence; likewise, the influence of the national research institutions is not supported by an appropriate position in the network. The lack of clear, shared knowledge of the whole governance framework is also testified by the low percentage of the entire network perceived, the differences between influence values assigned by the groups of key informants (Table 4), and the difficulties in fully mastering even supposedly formal relationships (i.e. control links) and those strongly relevant in farmers’ decision-making (i.e. advice or money flows involving farmers). Overall clarity on governance structure and dynamics is therefore missing, although it is considered one of the key principles of good and adaptive water governance as it can affect all the processes requiring high coordination and networking (i.e. the information and advice flow or the authorisation and control system; OECD 2015).

From a relational perspective, groundwater governance mainly depends on knowledge dissemination and control-based strategies. Control-based approaches are often criticised due to both the negative effects on the social-environmental resilience and the lack of long-term benefits (Mazmanian and Kraft 2009; Cox 2016). Moreover, in our study control-based strategies are strongly limited by practical and economic issues because of the large number of farms, as reported in network narratives. The poor capacity to conduct systematic and widespread controls affects the effectiveness and credibility of this strategy in the stakeholders’ perception. Besides, it should be noted that the reduction of subsidies from the Common Agriculture Policy may impair the whole control system since controls are based on the information provided by farmers to obtain subsidies. As regards knowledge dissemination, the lack of advice flow in all networks, except in those drawn by farmers, indicates the failure to consider knowledge that originated in the industrial sector although it is strongly relevant in farmers’ decision-making. The tools chosen by trade unions and authorities to communicate the ND substantially differ from those commonly used by farmers. This underpins a poor consideration of the informality, frequency and clear consistency with production goals, which characterise reliable sources according to farmers (i.e. other farmers and retailers). Besides, agricultural high schools are completely marginal (Fig. 4), and the opportunity to promote long-term change in farmers’ attitudes is not exploited (McGuire et al. 2013).

## Management and governance recommendations

Firstly, detected trends highlight that reducing nitrate inputs is required to halt increasing concentrations as fast as possible in wells with exceeding threshold values, in intermediate and deeper aquifers (Mas-Pla and Menció 2018). Although the estimate of groundwater residence time will complement the evaluation of ND environmental performance, it is clear that an input reduction is urgently needed to recover the water quality. Moreover, the effect of the increasing water withdrawal from intermediate and deeper aquifers should be monitored as it can induce nitrate migration to these deeper aquifer units. To this end, the number of monitoring wells tapping the intermediate and deeper aquifers should increase. Water abstraction policies should thus be defined in accordance with the objectives and the nitrogen inputs established by the ND. Finally, even where overall stability has already been detected, in-depth trend estimations (i.e. taking into account distribution in both space and concentration classes) should be carried out, including and constantly monitoring wells with concentrations lower than 25 mg L^−1^. Although the ND currently allows these wells to have less intensive monitoring programmes (i.e. every 8 years; EU Commission 1991), our study confirms that they can provide useful information on possible unforeseen side effects of current regulations (e.g. manure relocation).

As regards manure, its relocation produced by the occurrence of different protection levels in neighbouring municipalities (i.e. NVZs and nNVZs) should be also monitored. In doing so, controls could be strengthened by cross-checking nitrogen input, and the contribution of agriculture to increasing trends, compared to other sources, could be better understood by enhancing the accuracy of nitrogen input estimates. Overall, the whole-territory approach adopted by other EU nations (Smith et al. 2007) would avoid this relocation of nitrate contamination compared to the discrete zones designation.

The desirable strengthening of the control-based strategies on which governance is strongly based, cannot disregard an increase in adaptive capacity. In fact, adaptive governance is required to deal with both the uncertainty and the speed of environmental changes, and potential modifications to regulations. As increasing adaptive capacity needs to pursue a real shift in farmers’ values (de Snoo et al. 2013), the way farmers select the sources of knowledge and the presence of multiple knowledge flow should be considered (Munoz-Erickson and Cutts 2016; Inman et al. 2018). In this respect, actors who interact with farmers locally are required, because of the effectiveness of frequent and informal relationships. This change in the current knowledge-transfer strategy would be also consistent with the necessity of new social learning spaces highlighted by Nguyen et al. (2014). The comparison between structural and perceived influence in the network allows us to identify some suitable actors, whose role can be improved in this respect (e.g. Water Consortia, ARPA). To reach different age groups, agricultural high schools should be included in the knowledge flow, for example through dissemination activities required by funded research and conservation projects.

Finally, effective and adaptive socio-environmental systems also need to go beyond the unclear and ill-defined idea of governance dynamics by the actors involved themselves. Management changes should hence be coupled with a greater awareness of the governance structure (i.e. clearness of roles and responsibilities), as it could enhance the legitimacy of leadership and improve relationships (Bodin and Prell 2011; Akhmouch and Correia 2016). Therefore, dissemination activities should not only include ‘which’ agricultural practices are required, but also ‘who’ is involved in governance processes and ‘how’. A simpler governance arrangement would strongly help this process.

It is worth mentioning, however, that constrains and opportunities emerged in this study may not fully represent the case of other European regions. This is due, on the one hand, to the site-specific nature of both hydrogeological and socio-relational dynamics. On the other hand, based on national variations in implementation and/or stakeholders involved, other parts or functioning of the ND may be described differently. Indeed, the network approach used in our study focused on identifying the most relevant features of the governance system, based on the perception of the actors involved. Therefore, the identification of all relevant elements of the impact of legislation should also include other case studies and other approaches.

Although other studies on ND went beyond an environmental or agronomic perspective, they only dealt with specific aspects of governance or management, namely the integration between local and scientific knowledge (Nguyen et al. 2014), the factors influencing farmers’ collaborative arrangements for manure exchange (Asai et al. 2014) and the use by farmers of tools for balanced fertilisation (Ravier et al. 2016). Differently, our study frames specific or local aspects influencing the ND implementation in the multi-relational context in which they occur. Thanks to this approach, the analysis of specific governance or management issues is improved by detecting otherwise hidden criticalities, precisely produced by the coexistence of several socio-relational dynamics in the governance system. In the Lombardy plain, for example, the necessity of both rethinking knowledge-transfer processes and thoroughly considering the way farmers select information and collaborations, also highlighted by previous studies, cannot ignore the need to consider (i) the solid relationship between farmers and industries, (ii) the relative importance of knowledge transfer and control activities and (iii) the definition of the adaptive capacity that we want to preserve or obtain, to achieve a fully resilient socio-environmental system.

In this light, a governance-oriented debate on ND is currently missing, although it could enhance the current knowledge on the Directive performance, at present only partially understood, thus hampering its environmental success. Therefore, we believe that Member States should be required to provide to the EU Commission an assessment of the governance dynamics supporting the Directive implementation together with environmental monitoring data.

## Electronic supplementary material

Below is the link to the electronic supplementary material.

Supplementary material 1 (PDF 573 kb)
