# Supplementary material for: EU Nitrates Directive, from theory to practice: Environmental effectiveness and influence of regional governance on its performance
Source: Ambio. 2019 May 21;49(2):504–16. doi: 10.1007/s13280-019-01197-8 (PMC6965048; doi:10.1007/s13280-019-01197-8)
Supplement: Supplementary file 1 — Supplementary material 1 (PDF 573 kb) [file 13280_2019_1197_MOESM1_ESM.pdf]

Ambio

Electronic Supplementary Material

This supplementary material has not been peer reviewed.

Title: EU Nitrates Directive, from theory to practice: environmental effectiveness and influence of regional governance on its performance

Trend analysis

The Mann Kendall-test (Mann, 1945; Kendall, 1955) has the advantage of not assuming any distribution for the data and it has similar power and efficiency to parametric methods (Serrano *et al.*, 1999; Batlle Aguilar *et al.*, 2007). Nevertheless, measurements need to be taken at regular intervals, which is not the case for the wells belonging to the regional monitoring data (ARPA dataset). These wells may have been sampled a maximum of four times per year. Therefore, available data was selected, based on (Hirsch *et al.*, 1991) suggestions, and a list of suitable wells obtained. Firstly, for each well we only selected the data collected between April and June and between October and December, due to the higher number of samples collected during these months. However, the lack of a seasonal effect between the two groups had previously been demonstrated, by means of a Wilcoxon rank-sum test (Kent and Landon, 2013). The wells were then selected by dividing the study period into three sub-periods (2006-2009, 2010-2013, 2014-2016) and the percentage of collected data with respect to the number of data potentially available for each sub-period was calculated, considering an average data collection of 2 samples per year. Only wells with a higher coverage than 25% in each sub-period were included in the analysis. Data from 162 wells were hence found to be suitable for trend analysis (118 in SA, 31 in IA and 13 in DA), for a total number of 2907 data (approximately 18 measurements per well over the 11 year period considered).

All the analyses were performed using the statistical software Rstudio version 1.0.153 (RStudio and Team, 2015) and the packages *imputeTS* (Moritz, 2017) and *trend* (Polhert, 2017).

Social network analysis

The Net-map method is based on a participatory approach, which contributes to the in-depth understanding of both the dynamics at stake within a studied network and the differences in perspectives of each stakeholder (Bodin and Crona, 2009; Fuhse and Mutzel, 2011; Hauck *et al.*, 2015; Newing, 2010; Sayles and Baggio, 2017).

Firstly, a list of potential interviewees was drafted. We based our selection on (i) the level of involvement in the decisional and management processes concerning the studied issue, (ii) their level of expertise in their fields, (iii) the practical possibility of contacting and meeting them. Then we invited them by email and phone. This list included: 7 authorities (members of the Regional General Directorates for Agriculture and for Environment, and of ARPA), 2 farmers, 2 breeders, 6 organisations (representative of farmers' trade unions, environmental organizations, water consortia, natural parks), 7 scientists actively involved in research projects. The number of farmers and breeders was strongly limited by their difficulties in attending the meeting. All the authorities, farmers, breeders and researchers, and 2 organizations we invited, attended the meetings.

During the focus groups, each group of key informants was asked to draw an Influence Network Map (INM; Schiffer and Hauck, 2010). The guiding questions for creating the INMs were: "Who can influence groundwater pollution reduction in the Lombardy plain?" and "Who can influence the implementation of new groundwater protection actions based on the outcomes of the hydrogeological and biogeochemical investigation?". Basically, the interviewees were first asked to list all the actors involved in groundwater nitrate contamination and in ND application (e.g. contractors, consultants, consortia, breeders, citizens) for each category of actors (i.e. authorities, individual actors, research institutions, industries, organisations), and then to depict all the links between the actors for each category of link (i.e. authorisation and control, technical information,

advice and best practices, money, conflicts; Table S1). The categories of actors and links were previously defined by the researchers, with the main aim of facilitating and inspiring the discussion. In fact, it allowed the interviewees to orient themselves in a wide and varied governance context. At the same time, no further description of links was provided, compared to Table S1, as we also aimed to gain a deep understanding of the meanings that each group associate to the governance dynamics. Finally, the interviewees were asked to quantify the potential influence of each actor, according to their perception. This was evaluated on an influence-value scale, from 1 to 5, where 1 is the lowest influence level and 5 is the highest (decimal values were permitted). Description of the role of the actors, relationships and reasons for the influence values were encouraged, both in order to validate the information and to obtain qualitative data. When disagreement on effective involvement of an actor, link or on influence values occurred, the interviewees were asked to discuss the matter further until a consensus was reached within the group. Each focus-group took between 2-3 hours.

Before merging the 5 networks into a single INM, we assessed the relevance of (i) the stakeholders not listed by all groups of key informants, (ii) those whose function had not been clearly explained by the interviewees and (iii) the actors whose role was differently described by the groups of key informants. To this end, we used the information available in official documents and on web sites (i.e. documents provided by the regional, provincial or municipal authorities). Neither the actors who were not involved in the studied issue nor those who were only marginally involved (e.g. fish farmers or health authorities) were included in the final network. When a stakeholder was not listed by all groups and its role in official documents and web sites could not be verified, it was only included in the final INM if it was mentioned by someone with whom the stakeholder shares a relational link (i.e. the brokers who were mentioned by the farmers). The members belonging to the same institution or organisation and those whose difference in role and functions were not relevant to the studied regional scale, were merged to create the final INM (e.g. all the Italian ministries, the European Commissions or large/small-scale farmers/breeders). The same approach was also applied

for the links. If (i) a link was not listed by all groups of key informants, (ii) the description provided was not clear or (iii) the link was described differently by the groups of key informants, the link was verified by means of official documents and web sites. If it was an informal link, it was only included in the final INM when it was mentioned by key informants directly involved in the same relationship (i.e. a link listed and adequately described by the farmers, in which the same farmers were involved). When there was discrepancy on informal relationships between groups of key informants (e.g. in the case of the advice links), a conservative approach was applied and only ties with a satisfactory convergence of views were included in the final network. Moreover, a few days after the interview, the network produced by each group was shown to the key informants involved so that they could make changes and in order to share additional remarks. All deletions and changes made to the list of actors initially compiled by the key informants are reported in Table S3.

The average influence was calculated by also considering when actors were not listed by all focus-groups (i.e. considering an influence value equal to zero for each network in which that actor was not mentioned).

The social network was displayed and analysed using the software Visone (Brandes and Wagner, 2004).

Tables

Table S1. Description of links provided to interviewees.

Link	Description
Authorisation and control	Ties representing control activities on groundwater use and nitrogen input, the authorisation of activities which can cause groundwater contamination and the definition of the limits to these activities.
Technical information	Ties representing dissemination of practical information related to groundwater and fertiliser use.
Advice and best practices	Ties representing dissemination of practical or theoretical knowledge aimed to improve groundwater and fertiliser use.
Money	Ties representing the exchange of money directly or indirectly influencing groundwater use and nitrogen inputs.
Conflicts	Ties representing practical or theoretical disagreements or incompatibility that can influence groundwater or fertiliser use.

Table S2. Actors reported by the five focus groups. The number of actors mentioned and also included in the final INM is reported in brackets.

**Focus group 1 (20)**
Farmers
Breeders
ARPA
Farmers' trade unions
Breeders' trade unions
Environmental NGO
Health agency
Citizens
National Research Institute
EU Commission
Municipalities
Agricultural consortia
Water Consortia (Irrigation)
Water Consortia (Lakes)
Consumers
CREA (Research Institute)
DG Agriculture
DG Enviroment
Agricultural districts
International research institutes
ERSAF (research institute)
Water suppliers
Large breeders
Food industries
ISPRA
Fish farmers' trade unions
Fish farmers'
JRC
Large farmers'
Ministry of agriculture
Ministry of environment
Ministry of economy
Parks
Provinces
CRPA (research institute)
Universities

**Focus group 2 (17)**
Farmers
Breeders
Farmers' trade unions
Breeders' trade unions
Health agency
Citizens
EU Commission
Water Consortia (Irrigation)
Non-food companies
Food companies
Agricultural High Schools
Research Institute
Biogas producers
Fertilizers producers
Provinces
Lombardy region
National government
Agricultural consultants
Universities

**Focus group 3 (29)**
Farmers
Breeders
ARPA
Farmers' trade unions
Breeders' trade unions
Environmental NGO
Health agency
River basin authority
Environmental police
Agricultural trade unions' offices
Citizens
CNR (National research institute)
Municipalities
Industries' trade union
Water Consortia (Irrigation)
Water Consortia (Lakes)
Contractors
CREA (Research Institute)
EU Commision - agriculture
DG Agriculture
EU Commision - environment
DG Enviroment
EU Commision - Health
DG Health
Agricultural districts
ERSAF (Regional research institute)
Sludge Treatment Companies
Civil wastewater treatment plants
Agricultural machinery manufacturers
Food companies
ISPRA
JRC
Ministry of Health
Ministry of agriculture
Ministry of environment
Agricultural consultants' trade unions
Parks
Biogas producers
Fertilizers' producers
Provinces
Agricultural retailers
Agricultural consultants'
Water companies (civil)
Universities

**Focus group 4 (19)**
Farmers
Breeders
ARPA
Farmers' trade unions
Environmental NGO
River basin authority
Citizens
EU Commission
Municipalities
Water Consortia (Irrigation)
Water Consortia (Lakes)
Parks
Provinces
Lombardy region
Agricultural consultants
Universities
Environmental volunteers
Civil wastewater tr. plants
Industrial wastewater tr. plants
CNR
Foundations
Hydroelectric power plants

**Focus group 5 (15)**
Farmers
Breeders
ARPA
CIA (farmers' trade union)
Irrigation water consortia
Contractors
Food companies
Agricultural high schools
Brokers
Seeds companies
Biogas producers
Provinces
Lombardy region
Agricultural retailers
Universities
Compost producers
Fertiliser producers
National institute for rice cultivation
COLDIRETTI (farmers' trade union)
CONFAGR.farmers' trade union)

Table S3. List of the actors not included in the final INMs or not included as mentioned by the key informants.

**Actor**	**Notes**
Large-scale breeders	included in breeders
Consumers	included in citizens
Water suppliers	included in CWP
EU agriculture	included in EU
EU environment	included in EU
EU health	included in EU
CIA	included in FTU
Agricultural assistance centre	included in FTU
COLDIRETTI	included in FTU
CONFAGRICOLTURA	included in FTU
JRC	included in IRI
Ministery of health	included in NG
Ministry of agriculture	included in NG
Ministry of environment	included in NG
Ministry of economic development	included in NG
ERSAF	included in NRI
National Research Council	included in NRI
Rice Association	included in NRI
CREA	included in NRI
CRPA	included in NRI
ISPRA	included in NRI
Universities	included in NRI
Fertiliser companies	included in SFC
Seed companies	included in SFC
Water management office	included in SRA
Large-scale farmers	included in FRM
Health Agency	not relevant

Regional DG Welfare	not relevant
Agricultural consultants' associations	not relevant
Breeders' trade unions	not relevant
Fish farmers' unions	not relevant
Fish farmers	not relevant
CONFINDUSTRIA	not relevant
Agricultural districts	not relevant
Compost producers	not relevant
Hydroelectric power plants	not relevant
Regional administration	reported as DGA, DGE

Figures

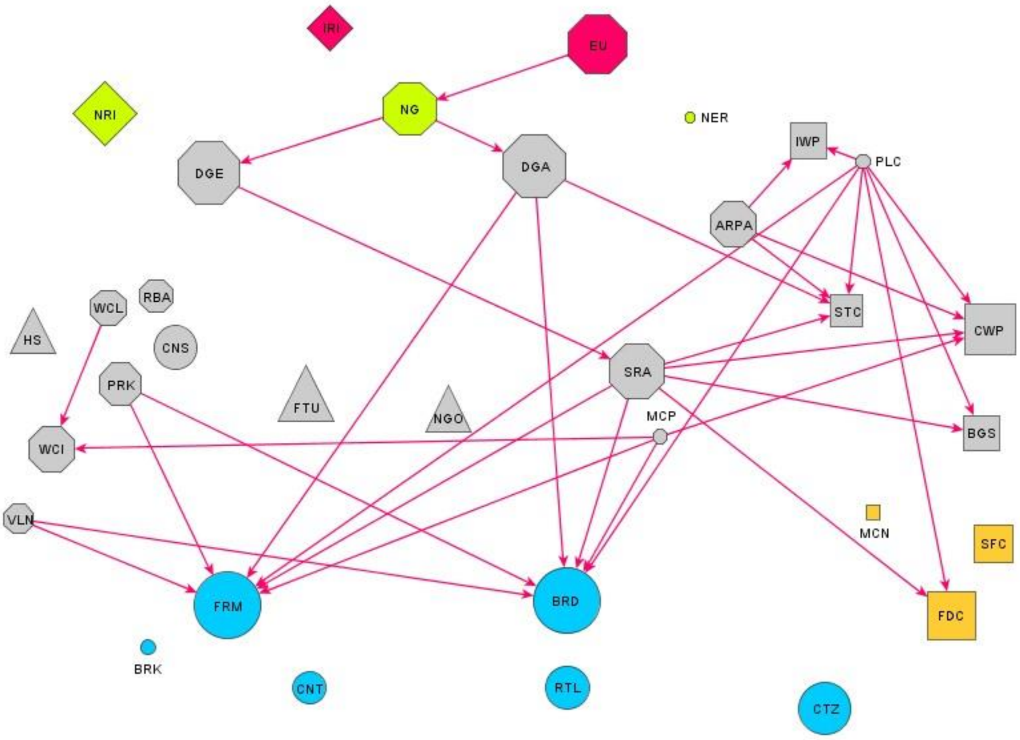

Figure S1. Authorisation and control links in the governance framework related to groundwater contamination in the Lombardy plain. The colours of the nodes correspond to the levels of governance; pink: international, green: national, grey: sub-national, light blue: local. Orange nodes represent multilevel actors. The size of the nodes corresponds to the perceived influence of each actor.

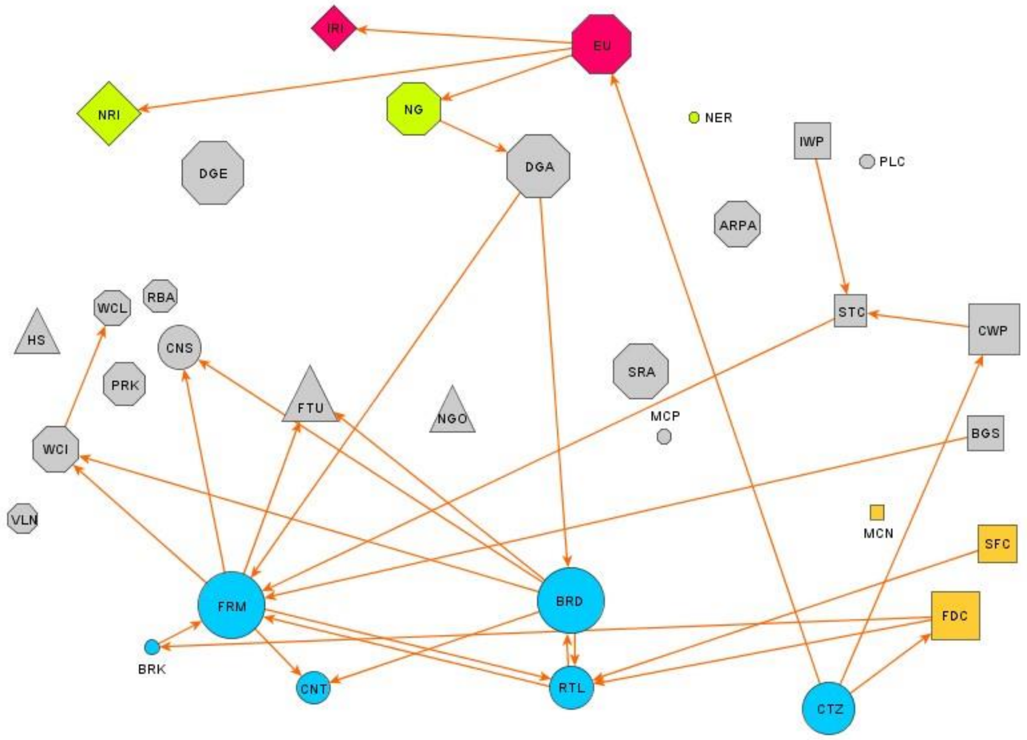

Figure S2. Money links in the governance framework related to groundwater contamination in the Lombardy plain. The colours of the nodes correspond to the levels of governance; pink: international, green: national, grey: sub-national, light blue: local. Orange nodes represent multilevel actors. The size of the nodes corresponds to the perceived influence of each actor.

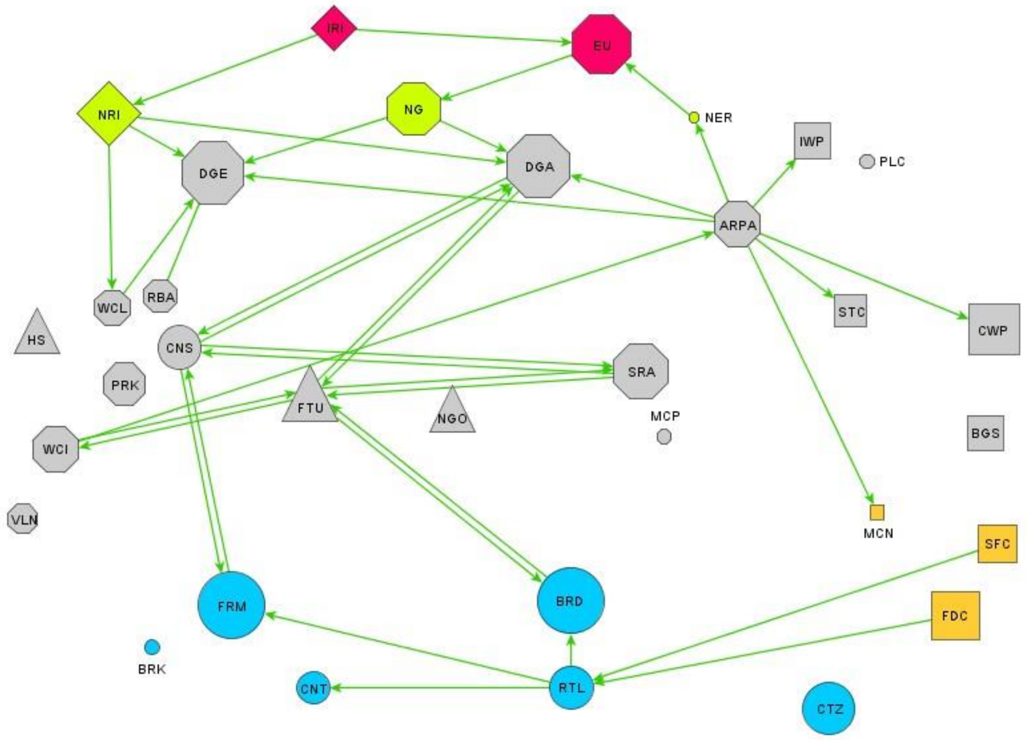

Figure S3. Technical information links in the governance framework related to groundwater contamination in the Lombardy plain. The colours of the nodes correspond to the levels of governance; pink: international, green: national, grey: sub-national, light blue: local. Orange nodes represent multilevel actors. The size of the nodes corresponds to the perceived influence of each actor.

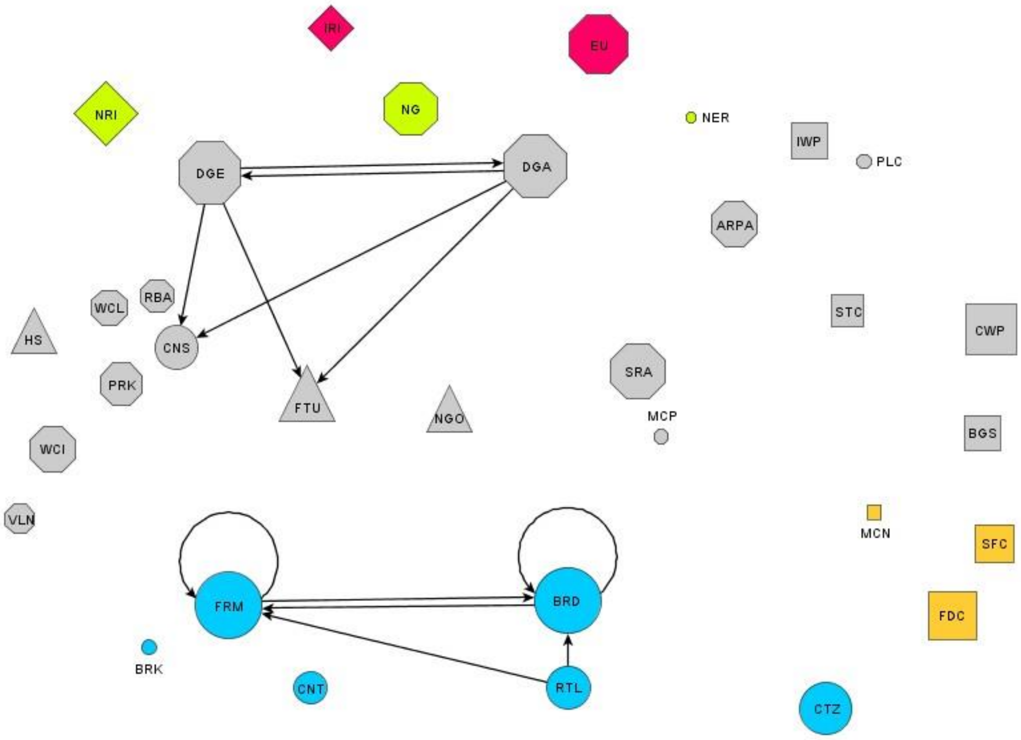

Figure S4. Advice links in the governance framework related to groundwater contamination in the Lombardy plain. The colours of the nodes correspond to the levels of governance; pink: international, green: national, grey: sub-national, light blue: local. Orange nodes represent multilevel actors. The size of the nodes corresponds to the perceived influence of each actor.

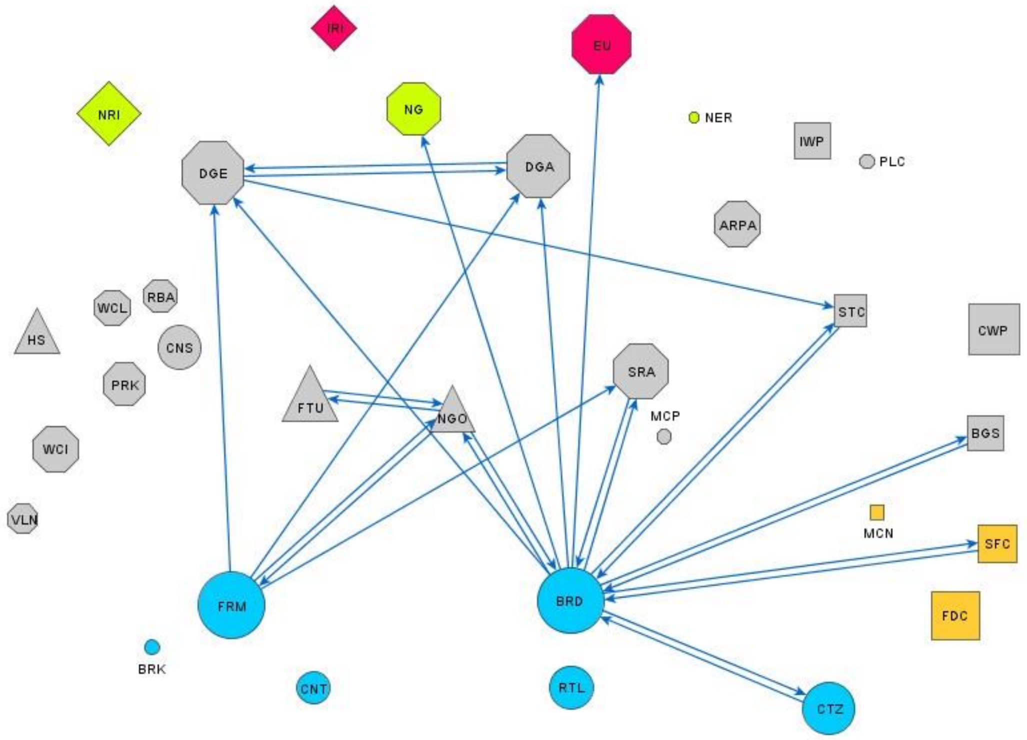

Figure S5. Conflict links in the governance framework related to groundwater contamination in the Lombardy plain. The colours of the nodes correspond to the levels of governance; pink: international, green: national, grey: sub-national, light blue: local. Orange nodes represent multilevel actors. The size of the nodes corresponds to the perceived influence of each actor.

References

Batlle Aguilar J, Orban P, Dassargues A and Brouyere S 2007 Identification of groundwater quality trends in a chalk aquifer threatened by intensive agriculture in Belgium *Hydrogeology Journal* **15** 1615-27

Bodin O and Crona B I 2009 The role of social networks in natural resource governance: What relational patterns make a difference? *Global Environmental Change-Human and Policy Dimensions* **19** 366-74

Brandes, U., and D. Wagner. 2004. Analysis and visualization of social networks. In *Graph drawing software,* ed. M. Jünger, P. Mutzel, 321-340. Springer, Berlin, Heidelberg

Fuhse J and Mutzel S 2011 Tackling connections, structure, and meaning in networks: quantitative and qualitative methods in sociological network research *Quality & Quantity* **45** 1067-89

Hauck J, Stein C, Schiffer E and Vandewalle M 2015 Seeing the forest and the trees: Facilitating participatory network planning in environmental governance *Global Environmental Change-Human and Policy Dimensions* **35** 400-10

Hirsch R M, Alexander R B and Smith R A 1991 Selection of methods for the detection and estimation of trends in water-quality. *Water Resources Research* **27** 803-13

Mann, H.B. 1945. Nonparametric tests against trend. *Econometrica: Journal of the Econometric Society* 245-59

Moritz, S. 2017. imputeTS: Time Series Missing Value Imputation URL http://CRAN.R-project.org/ package=imputeTS. R package version 2.3.

Kendall, M.G. 1955. *Rank correlation methods*. Griffin, London.

Kent R and Landon M K 2013 Trends in concentrations of nitrate and total dissolved solids in public supply wells of the Bunker Hill, Lytle, Rialto, and Colton groundwater subbasins, San Bernardino County, California: Influence of legacy land use *Science of the Total Environment* **452** 125-36

Newing H 2010 *Conducting research in conservation: Social science methods and practice*: Routledge.

Polhert, T. 2017. Non-Parametric Trend Tests and Change-Point Detection. Version 1.0.0.

RStudio and Team. 2015. RStudio: Integrated Development for R. RStudio, Inc., Boston, MA URL http://www.rstudio.com/.

Sayles J S and Baggio J A 2017 Who collaborates and why: Assessment and diagnostic of governance network integration for salmon restoration in Puget Sound, USA *Journal of Environmental Management* **186** 64-78

Schiffer E and Hauck J 2010 Net-Map: Collecting Social Network Data and Facilitating Network Learning through Participatory Influence Network Mapping *Field Methods* **22** 231-49

Serrano A, Mateos V L and Garcia J A 1999 Trend analysis of monthly precipitation over the Iberian Peninsula for the period 1921-1995 *Physics and Chemistry of the Earth Part B-Hydrology Oceans and Atmosphere* **24** 85-90
